# Impact of new European Respiratory Society/American Thoracic Society bronchodilator reversibility interpretation on asthma diagnosis in children

**DOI:** 10.1002/ped4.70017

**Published:** 2025-07-16

**Authors:** Natalie Blyth, Joe Madge, Erol A. Gaillard, David K. H. Lo

**Affiliations:** ^1^ Department of Paediatric Respiratory Medicine University Hospitals of Leicester NHS Trust Leicester UK; ^2^ Department of Respiratory Sciences University of Leicester Leicester UK

Asthma is the most common chronic condition in children, affecting approximately 10%–15% of children worldwide.^1^ The disease, characterised physiologically by reversible airflow obstruction, is challenging to diagnose as a result of non‐specific and variable symptoms^1^ and poor access to objective lung function tests in most care settings. In children, asthma misdiagnosis has been reported in up to 54% of cases.^2^ Therefore, every major asthma guideline recommends the use of objective testing to improve the accuracy of diagnosis.

Demonstration of bronchodilator reversibility (BDR), using spirometry, following inhalation of a bronchodilator medication, is central to most asthma diagnostic criteria. Previous European Respiratory Society (ERS) and American Thoracic Society (ATS) spirometry interpretation guidelines, and most current asthma guidelines, recommend using a relative increase in absolute forced expiratory volume in 1 s (FEV_1_) and/or forced vital capacity (FVC) in litres of ≥12% and ≥200 mL from baseline as evidence of significant BDR (ERS2005).^3^ However, concerns of sex and size bias using this method have been raised; children who are smaller, with lower baseline lung volumes, will achieve a relatively larger % increase in FEV_1_ for any given increase in FEV_1_ in litres compared with larger/older children with higher baseline lung volumes.

Alternative calculations have been proposed which either report BDR as positive if the FEV_1_ (L) increase is ≥10% relative to the predicted value (ERS2021),^4^ or if the increase in FEV_1_ *z*‐score (*z*‐change)^5^ is ≥0.7. These methods are suggested to better account for differences in sex and size when interpreting BDR.

Two recent studies have compared BDR interpretation using either the older ERS2005 or newer ERS2021 methods.^6^, ^7^ In one study,^6^ 1365 tests in children with asthma or chronic cough aged 5–18 were compared. Approximately 5% of tests were reclassified using the more recent ERS2021 method; 4% from negative to positive, and 1% from positive to negative. The second study^7^ included 2293 paired pre‐ and post‐bronchodilator spirometry results from a population‐based cohort in Melbourne, Australia. All spirometry tests were performed as part of age 6‐year health assessments, so they did not include children from any other age group, but did include healthy children with no respiratory symptoms. Using the ERS2021 method, the number of positive BDR classifications more than doubled compared with using the ERS2005 calculation. Importantly, only 20% of children who were reclassified from BDR negative using ERS2005, to BDR positive using ERS2021 had symptoms suggestive of asthma. The authors concluded that the newer ERS2021 definition for increased BDR, which is based on adult data, has limitations when applied in young children and may contribute to over‐diagnosis of asthma.^7^

However, neither previous study has assessed the impact of height, weight, or sex on BDR interpretation, and neither has compared the alternative *z*‐change BDR calculation method using changes in *z*‐scores.

The aim of our study was to specifically explore how BDR classification would change in a cohort of children presenting with symptoms suggestive of asthma if existing (ERS2005) BDR calculation methods were changed to newer proposed methods (ERS2021, *z*‐change); and whether children of different height, weight and sex would be affected differently.

We performed secondary data analysis of lung function results obtained as part of a prospective primary care asthma study in children (NCT02913872), which was approved by a UK research ethics committee. Spirometry with BDR testing was available from 180 consecutively recruited children (aged 5–16 years) from primary care with symptoms suggestive of asthma, and performed in accordance with ERS/ATS standards. Participants performed spirometry pre‐ and post‐400 µg salbutamol by metered‐dose inhaler administered via a spacer. Written informed consent was obtained from all participating families. No participants had a prior formal diagnosis of asthma confirmed on objective testing. We calculated BDR for each participant using methods and thresholds recommended by ERS2005 (percentage change in FEV_1_ and/or FVC in litres from baseline; ≥12% and ≥200 mL increase from baseline is deemed positive),^3^ ERS2021 (change in FEV_1_ (L) relative to predicted; ≥10% increase is deemed positive),^4^ and *z*‐change (change in FEV_1_ *z*‐score; ≥0.7 increase in *z*‐score is deemed positive).^5^ BDR results were categorised as either positive (if BDR is higher than the recommended threshold) or negative (if BDR is lower than the recommended threshold) for each child using the calculation and cut‐off threshold recommended by each of the three methods.

We reported the number (%) of BDR results in all children, calculated using the more recent ERS2021 and *z*‐change methods, which were concordant or discordant with ERS2005 (the recommended method by most existing asthma diagnostic guidelines). We further reported the number (%) of concordant and discordant outcomes stratified by age, height, and sex, comparing ERS2021 and *z*‐change (Table 1) with ERS2005. The degree of agreement between methods was calculated using Cohen's kappa (*k*). Statistical analyses were performed using IBM SPSS Statistics (version 27).

**TABLE 1:** Bronchodilator response outcomes of ERS2021 and *z*‐change interpretation compared to ERS2005 interpretation

Alternative method	BDR results using ERS 2005^†^				Degree of agreement between methods: Cohen's Kappa (*k*)
	Concordant with alternative method [*n* (%)]		Discordant with alternative method [*n* (%)]		
	Positive BDR	Negative BDR	Positive BDR	Negative BDR	
**ERS 2021** ^‡^					
All children (*n* = 180)	45 (93.7)	125 (94.7)	3 (6.3)	7 (5.3)	0.86
Age (years)					
≥12	15 (83.3)	51 (98.1)	3 (16.7)	1 (1.9)	0.84
<12	30 (100.0)	74 (92.5)	0 (0)	6 (6.5)	0.87
Sex					
Male	31 (93.9)	81 (95.3)	2 (6.06)	4 (4.7)	0.88
Female	14 (93.3)	44 (93.6)	1 (6.67)	3 (6.4)	0.83
Height (cm)					
≥147	22 (88.0)	64 (98.5)	3 (12.0)	1 (1.5)	0.89
<147	23 (100.0)	61 (91.0)	0 (0)	6 (9.0)	0.84
***z*‐change** ^§^					
All children (*n* = 180)	42 (87.5)	105 (79.6)	6 (12.5)	27 (20.5)	0.59
Age (years)					
≥12	16 (88.9)	43 (82.7)	2 (11.1)	9 (17.3)	0.64
<12	26 (86.7)	62 (77.5)	4 (13.3)	18 (22.5)	0.56
Sex					
Male	29 (87.9)	68 (80.0)	4 (12.1)	17 (20.0)	0.61
Female	13 (86.7)	37 (78.7)	2 (13.3)	10 (21.3)	0.55
Height (cm)					
≥147	23 (92.0)	54 (83.1)	2 (8.0)	11 (16.9)	0.68
<147	19 (82.6)	51 (76.1)	4 (17.4)	16 (23.9)	0.50

Abbreviations: BDR, bronchodilator reversibility; ERS, European Respiratory Society.

^†^ Increase in absolute forced expiratory volume in 1 s (FEV_1_) and/or forced vital capacity (FVC) in litres of ≥12% and ≥200 mL.

^‡^ ≥10% change in FEV_1_ (L) relative to predicted.

^§^ Change in FEV1 *z*‐score of ≥0.7.

Compared with ERS2005, the ERS2021 method would have resulted in an additional 5% of children being reclassified as BDR‐positive and hence meet the threshold for an asthma diagnosis, and using *z*‐change, an additional 21% of children. Conversely, in children with a positive BDR according to ERS2005, 6% would have been reported as negative if using ERS2021, and 13% if using *z*‐change (Table 1). Overall, agreement between ERS2005 and ERS2021 was good (*k* = 0.86), and between ERS2005 and *z*‐change was moderate (*k* = 0.59).

When stratified by age, height, and sex, agreement between ERS2005 and ERS2021 was substantial‐to‐good, with the lowest agreement between methods seen in females (*k* = 0.83) and the highest in children ≥147 cm tall (*k* = 0.89).

Comparing ERS2005 with *z*‐change, the lowest agreement was observed in children <147 cm (*k* = 0.50) and in females (*k* = 0.55), whilst the highest agreement was observed in children ≥147 cm tall (*k* = 0.68).

We observed clinically important differences in the reporting of BDR depending on the method used to calculate and interpret the change in FEV_1_ pre‐ and post‐bronchodilator. Similar to previous studies,^6^, ^7^ we observed a significant number of BDR results being reclassified in children within our cohort using alternative calculation methods. Compared with the ERS2005 method, changing to either the ERS2021 or *z*‐change methods would potentially result in between 6% and 18% of children having their asthma diagnosis re‐categorized.

Our study had limitations, including a relatively small sample size and no availability of additional objective test data (such as bronchial provocation tests) to confirm an asthma diagnosis. However, we have added to the existing literature through the stratification of our analysis by age, size, and sex to explore whether there was evidence of size/sex bias when reporting BDR by different methods. We observed the poorest agreement in BDR interpretation using the various calculation methods in children <12 years of age, female, or <147 cm tall. These groups of children are potentially at higher risk of asthma misdiagnosis depending on the method of calculation used. Moreover, unless there is consensus for all asthma diagnostic guidelines to change their recommendations in parallel, asthma diagnosis may differ between local, national, and international centres depending on which recommendation is followed.

Existing asthma diagnostic guidelines have been based on evidence from studies that have used the ERS2005 BDR calculation method as the gold standard for asthma diagnosis. A change to newer calculation methods will not only impact asthma diagnosis in individual children moving forward, but also bring into question the reliability of other asthma objective tests, which have been validated using BDR calculated using the ERS2005 method as the gold standard. Pediatric‐specific studies are needed to determine which BDR calculation method is the most accurate for confirming asthma in children, using an alternative accepted objective method as a gold standard (such as bronchial provocation testing).

In conclusion, we have identified important and clinically relevant implications for asthma diagnosis in children based on the method and threshold used to calculate and interpret BDR. Age, sex, and height‐specific population‐level data are needed to determine the “normal” range for bronchodilator reversibility in children and young people to improve the accuracy of detection of abnormal bronchodilatory response. Importantly, any changes to recommendations for BDR calculations should be made with careful consideration of their clinical impact on patients and coordinated with clinical asthma guidance.

## ETHICAL APPROVAL

Ethics approval was obtained from the NHS research ethics committee (16/EM/0162).

## CONFLICT OF INTEREST

David K. H. Lo receives support from the National Institute for Health and Care Research (NIHR) advanced fellowships. David K. H. Lo has also received institutional research grants from the Midlands Asthma and Allergy Research Association (MAARA), NIHR, and the Small Business Research Initiative (SBRI), and travel expense payments from the European Respiratory Society (ERS). Erol A. Gaillard has received institutional grants from Gilead, Chiesi, Propellar Health, Helicon Health, Adherium Ltd, and AZ; travel expense payments from ERS; and personal fees from Circassia and Sanofi. NB and JM had no competing interests to declare.
